# Effect of Zinc Hydroxychloride and Copper Hydroxychloride in Compound Feed on Rearing Results and Carcass Characteristics of Broiler Chickens

**DOI:** 10.3390/ani16132059

**Published:** 2026-07-03

**Authors:** Sabina Kaim, Dorota Banaszewska, Barbara Biesiada-Drzazga

**Affiliations:** Institute of Animal Science and Fisheries, University of Siedlce, Prusa 14, 08-110 Siedlce, Poland; s.kaimmirowski@gmail.com (S.K.); barbara.biesiada-drzazga@uws.edu.pl (B.B.-D.)

**Keywords:** zinc, copper, broiler chickens, compound feed, production results, micronutrient bioavailability, meat quality

## Abstract

The aim of the study was to compare the production results, slaughter value, and physicochemical and biochemical parameters of the muscles of Ross 308 broiler chickens. The study included 225 individuals divided into three groups of 75 birds each. Each group was subdivided into three subgroups of 25 birds each (replicates). The control group received zinc (Zn) in the form of zinc oxide (ZnO) and copper (Cu) in the form of copper (II) sulfate pentahydrate (CuSO_4_·5H_2_O). Experimental group I received the microelements zinc and copper exclusively in the form of zinc hydroxychloride and dicopper chloride trihydroxide. Experimental group II received zinc and copper in both inorganic and hydroxychloride forms. The obtained results were processed using statistical analysis using the STATISTICA 13.0 [2016] program. The significance of differences between groups was inferred based on Tukey’s test. Chickens from the experimental groups were characterized by higher body weight (*p* ≤ 0.05) and eviscerated carcass weight (*p* ≤ 0.05) and better feed utilization (*p* ≤ 0.05). Moreover, compared with the control group, the carcasses of chickens from the experimental groups were characterized by a lower weight of breast muscles (*p* ≤ 0.05) and skin with subcutaneous fat, a similar weight of leg muscles, and a significantly greater weight of the remaining carcass components (*p* ≤ 0.01). The use of Zn and Cu in the form of hydroxychlorides in broiler chicken nutrition increased the fat content in the muscles (*p* ≤ 0.05), the concentrations of Zn and Cu in the liver, and significantly improved the tibial bone strength of broiler chickens. The application of Zn and Cu in the form of hydroxychlorides in the diets (experimental groups I and II) enhanced lipid and protein oxidation processes and the overall antioxidant capacity in the breast muscle. To conclude, dietary supplementation with zinc and copper hydroxychlorides in broilers promotes growth performance, bone strength, and mineral bioavailability, though it reduces breast muscle yield and triggers oxidative stress in these tissues.

## Simple Summary

In recent years, various feed additives have been used in broiler chicken nutrition to improve rearing efficiency. Both zinc (Zn) and copper (Cu) play a key role in numerous physiological functions, metabolic processes, and the proper functioning of the immune system. The authors of this study have demonstrated that the use of microelements in the form of hydroxychlorides increases the body weight (BW) of broiler chickens and results in better feed conversion. An influence of the administered form of Zn and Cu was observed on the weight and tissue composition of the carcass, as well as on the crude fat content in the analyzed muscles. Additionally, it was determined that using forms of elements with higher bioavailability affects the activity of antioxidant enzymes and significantly improves the strength of the tibia bone. The thematic scope of this research is of great importance, as providing broiler chickens with specific forms of Zn and Cu microelements can contribute to the improvement of production efficiency.

## 1. Introduction

In the process of rational animal nutrition, the current aim is to achieve desired production effects while simultaneously maintaining the lowest possible expenditures on feed through the use of effective nutrition technologies [1]. In the nutrition of broiler chickens, various feed additives are used to improve rearing efficiency [2,3,4,5], including the incorporation of micro- and macroelements—specifically zinc (Zn) and copper (Cu)—into feed mixtures [6,7].

Zinc (Zn) plays a key role in many physiological functions of the organism, including the functioning of the immune system and the development of cells, tissues, and bones, as well as the metabolism of proteins, carbohydrates, and lipids [8]. Zinc serves to regulate the functioning of various enzymatic systems in the bird’s body [9]. However, excessive administration of Zn can lead to toxicity and negatively affect the health of chickens [10].

In turn, copper (Cu) is essential for the positive course of numerous metabolic processes in broiler chickens, including the proper functioning of the immune system, the production of red blood cells, and the maintenance of healthy bones and tissues. It has been shown that high dosing of Cu affects the structure, function, and profile of the intestinal microbiome [11]. The beneficial effect of Cu supplementation on micro-openings in the gastrointestinal tract and its bactericidal or bacteriostatic action has been demonstrated in many studies [12,13,14]. Thanks to this, the appropriate amount in feed mixtures can reduce the birds’ susceptibility to diseases by decreasing the recruitment and infiltration of intestinal lymphocytes, thereby increasing the absorption of nutrients [15]. Kwiecień and Winiarska-Mieczan [16] confirm clear benefits from the use of copper sulfate pentahydrate (CuSO_4_·5H_2_O) in chicken nutrition. According to Wu et al. [17], the addition of (CuSO_4_·5H_2_O) to feed intended for broiler chickens positively influenced their growth, performance, and health status. It should be noted, however, that an excessive amount of Cu can adversely affect the organism and even show toxic effects [10,18,19,20,21]. The studies cited above show that most often the source of origin of Cu and Zn, rather than their quantity, decisively influences the production indices of poultry.

Despite the vast diversity of Cu and Zn forms approved for use in animal nutrition, the most popular are: inorganic zinc oxide (ZnO) and (CuSO_4_·5H_2_O) as well as organic forms of glycine chelates of both these elements [22]. However, the use of hydroxychloride forms of microelements, despite the safety of their use approved by the European Union, is still not very popular (Commission Implementing Regulation (EU) 2016/673 of 29 April 2016). In numerous experiments, a positive response of chickens to Zn and Cu supplementation from various sources has been noted [15,23,24], while in others, minimal or no influence was obtained [25,26].

The aim of the study was to compare the production results, slaughter value, and physicochemical and biochemical parameters of the muscles of broiler chickens fed throughout the entire rearing period with feed mixtures containing different forms of Zn and Cu.

## 2. Materials and Methods

The research material consisted of 225 broiler chickens of the Ross 308 set. The broiler chickens were kept in standard environmental conditions, on litter, with a stocking density of 14 pcs/m^2^ (https://aviagen.com/assets/Tech_Center/Ross_Broiler/RossxRoss308-BroilerPerformanceObjectives2022-EN.pdf). One-day-old chicks were weighed in groups and marked individually with wing tags, and then the birds were randomly assigned to three research groups. Each group numbered 75 individuals. Each group was subdivided into three subgroups of 25 birds each (replicates). The birds were reared until the age of 42 days. The experimental design is presented in Table 1.

Throughout the entire period of the experiment, the broiler chickens were fed ad libitum with complete feed mixtures in the form of crumbles. The mixtures contained amounts of nutrients consistent with the poultry feeding recommendations [27] (Table 2).

The microelements contained in the feed were inorganic in nature. The control group received Zn and Cu in inorganic forms contained in the mineral-vitamin supplement. The experimental groups received feed in which Zn and Cu were in hydroxychloride forms. Experimental group I received the microelements Zn and Cu exclusively in the form of hydroxychlorides. Experimental group II received Zn and Cu in both inorganic and hydroxychloride forms (Table 3).

Throughout the entire duration of the experiment, the amount of feed provided and uneaten residues were recorded for each group. This allowed for the calculation of feed consumption per bird per 1 kg of body weight gain. Feed intake was calculated as the difference between the amount of feed offered to the birds during a given week and the amount of feed remaining uneaten. Based on weekly BW measurements of the broiler chickens, weekly body weight gain was determined. The ratio of feed intake to body weight gain during a given week was used to calculate the feed conversion ratio (FCR). Furthermore, bird mortalities and health culls were recorded on an ongoing basis. Data obtained from the results of BW, feed consumption, and chicken mortality during the rearing period served to calculate the European Production Efficiency Factor (EPEF) rearing efficiency index for each group of broiler chickens [28]. The EPEF was calculated according to the formula: EPEF = (average BW (kg) × survivability (%)/rearing days × feed intake per kg of BW) × 100.

After the completion of rearing, 25 birds were randomly selected from each of the groups for slaughter. In total, 75 chickens were slaughtered. The slaughter was carried out under production conditions in a poultry slaughterhouse, and the slaughter analysis and biochemical analyses under laboratory conditions. After slaughter, bleeding, and plucking, a slaughter analysis was performed according to the methodology given by Ziołecki and Doruchowski [29]. On an electronic scale, offal (heart, liver, and gizzard), inedible viscera (including the head), and shanks were weighed and then the slaughter yield of the chickens was determined. Slaughter yield was calculated as the ratio of carcass weight to pre-slaughter BW, expressed as a percentage. Individual tissue components were separated from the carcass, then weighed, and their share in the carcass mass was determined.

In order to determine the content of Zn and Cu in the liver, the method described by Gajula et al. [30] was used. After preparation, the liver sample was analyzed for Zn and Cu content using an atomic absorption spectrophotometer (Varian, SpectrAA-220FS, SpectraLab, Scientific Incorporation, Amherst, NY, USA) at wavelengths of 213.9, 279.5, 324.7, and 248.3 nm, respectively.

After evisceration, the carcasses were cooled at a temperature of about 8–10 °C, and after 24 h, they were divided into tissue components. From each carcass, the skin with subcutaneous fat, breast muscles (superficial and deep), leg muscles (thighs and drumsticks), and the remainder of the carcass (bones and remaining muscles) were separated.

From each carcass, samples of breast muscles and leg muscles (thighs and drumsticks) were taken to determine physicochemical parameters: dry matter, total protein, ash, and crude fat using a near-infrared spectroscopy (NIR. FOSS, Hillerød, Denmark) apparatus. The NIR technique analyzes agricultural and food products based on the natural electromagnetic spectrum in the wavelength range from 700 to 2500 nm.

Furthermore, biochemical studies were carried out on the breast muscle, including the determination of antioxidant potential (AOP) content, activity of enzymes from the glutathione peroxidase (GPx) family, superoxide dismutase (SOD), free radical scavenging (DPPH), glutathione (GSH), malondialdehyde (MDA), and carbonyl groups. The antioxidant activity of the analyzed material was carried out according to the modified procedure of Brand-Williams et al. [31]. Furthermore, strength tests of the tibia bones were carried out. The average load at fracture (N) was determined using an INSTRON 3345 (INSTRON, Norwood, MA, USA) apparatus using Bluehill Universal (version 4) software simulating bone loading with a BEND FIXTURE 10 mm ANVIL attachment.

The obtained results were processed using statistical analysis using the STATISTICA 13.0 [2016] program. Mean values and standard deviation were determined. The significance of differences between groups was inferred based on Tukey’s test (a, b—significant differences between groups at *p* ≤ 0.05; A, B—significant differences between groups at *p* ≤ 0.01).

## 3. Results

In the first 21 days of rearing, the body weight of the chickens was similar in all research groups (Table 4), and above this age, until the end of rearing, the chickens from experimental group I receiving Zn and Cu elements in the form of highly absorbable hydroxychlorides and from experimental group II receiving mixtures containing this form of Zn and Cu in both inorganic and hydroxychloride form, achieved a BW greater by 100–120 g than the chickens from the control group (*p* ≤ 0.05).

No influence of the applied complete feed mixture on the mortality of chickens during the rearing period was shown (Table 5). For the entire rearing period, this index was within the limits of 3.14–3.51%. The average consumption per 1 kg of BW gain amounted to 1.61 kg in the control group, and in experimental groups I and II, 1.55 kg and 1.57 kg, respectively. Diverse values regarding the final BW of the chickens and feed consumption per 1 kg of BW gain by the birds, with a fairly similar level of survival and the same length of rearing, influenced the diversification of the EPEF index. Its highest value was obtained in experimental group I, an intermediate value in experimental group II, and the lowest in the control group. Statistical differences were confirmed between the control and experimental groups 1 (*p* ≤ 0.05).

The average BW of the chickens intended for slaughter oscillated between 2556 g (in the control group) and 2679 g (in experimental group I) (Table 6). The highest, statistically confirmed BW before slaughter and weight of the eviscerated carcass were characterized by the chickens from experimental group I, receiving Zn and Cu in the complete feed mixture exclusively in the form of hydroxychlorides; slightly lower by the chickens from experimental group II, receiving Zn and Cu in the complete feed mixture in both inorganic and hydroxychloride form (of varied bioavailability); and the lowest by the chickens from the control group, fed with mixtures containing Zn and Cu in inorganic forms contained in the mineral-vitamin supplement.

Significant diversification of the slaughter analysis results of broiler chickens in the weight of the eviscerated carcass reflected the differences in individual tissue components of the carcass in the research groups of chickens (Table 7). Despite the significantly lower weight of the eviscerated carcass in the control group chickens, it was found to be characterized by a higher weight of breast muscles, including the weight of deep muscles, a higher weight of skin with subcutaneous fat (*p* ≤ 0.05), and a significantly lower weight of the remainder of the carcass compared to the experimental groups (*p* ≤ 0.05; *p* ≤ 0.01). The chickens from experimental group I, receiving microelements in the form of hydroxychlorides, were characterized by a highly significantly higher weight of bones and remaining muscles in the carcass (827.01 g) compared to the control group (691.20 g) (*p* ≤ 0.01). Statistical differences at the level (*p* ≤ 0.05) were confirmed between all groups. The observed differences suggest a significant influence of the form of administered microelements on the skeletal development of broiler chickens.

A comparison of the tissue composition of the chicken carcasses from individual groups indicates that diversified nutrition affects not only the BW of the chickens but also the tissue composition of the carcass (Table 8). Depending on the applied form of Zn and Cu in the mixtures, carcasses differing in terms of the content of individual tissue components were obtained.

The share of breast muscles in the examined carcasses ranged from 28.20 to 32.39% (*p* ≤ 0.05), leg muscles from 21.98 to 22.44%, skin with subcutaneous and abdominal fat from 9.25 to 9.93%, and the remainder of the carcass (bones and other muscles) from 35.23 to 40.57% (statistically confirmed differences). Significantly lighter carcasses of chickens from the control group were distinguished by a better tissue composition than the carcasses from experimental groups I and II. This was expressed by clearly better muscularity, especially of the breast, similar fatness, and a significantly lower share of the remainder of the carcass (bones and other muscles). On the other hand, the carcasses of chickens from the experimental groups were characterized by a fairly similar share of tissue components; however, chickens from experimental group II, receiving Zn and Cu in mixtures in both inorganic and hydroxychloride form, compared to chickens from experimental group I, receiving the aforementioned elements in the form of highly absorbable hydroxychlorides, were characterized by slightly better muscularity and a lower share of the remainder of the carcass (bones and other muscles).

Chickens fed with microelements in the form of hydroxychlorides showed a higher share of crude fat in the breast and leg muscles, as well as a higher ash content in the leg muscles (crude ash level) compared to the control group (statistically confirmed differences) (Table 9). It can therefore be assumed that the use of hydroxychloride forms of Zn and Cu in feed modifies the parameters of the chemical composition of muscles.

The activity of antioxidant enzymes and DPPH, which serve as indicators of biological reactivity, is controlled by the levels of Zn and Cu in the body. The use of element forms with higher bioavailability can significantly influence the functioning of these enzymes. The results of measurements of antioxidant enzyme activity and DPPH in the breast muscles of chickens indicate significant differences between the research groups (Table 10). The studies confirmed a significant effect of higher bioavailability of Zn and Cu on the activity of GPx compared to the control group. GPx activity was significantly higher in experimental group I (152.873 nmol/min/mL) compared to the control group (128.773 nmol/min/mL) and experimental group II (136.387 nmol/min/mL) (*p* ≤ 0.05). Additionally, the concentration of carbonyl groups was significantly higher in experimental group I (45.282 nmol/mL) compared to the control group (34.499 nmol/mL) and experimental group II (39.394 nmol/mL). The level of GSH activity, a protein that neutralizes oxygen-free radicals and counteracts oxidative stress, differed significantly between the experimental groups. The GSH level was significantly higher in experimental group I (30.482 µM-SH) compared to the control group (19.365 µM-SH) and experimental group II (24.188 µM-SH). The higher GSH level in experimental group I suggests a clear influence of Zn and Cu hydroxychloride on this parameter, which may contribute to reducing immunosuppression in the animal’s body. The concentration of MDA was significantly higher in experimental group I (0.45 µM) compared to the control group (0.346 µM) and experimental group II (0.42 µM). The observed significant increase in the MDA level in the breast muscles of chickens from experimental group I indicates an intensification of cellular oxidative stress compared to the control group, which showed the lowest MDA concentration. The analysis of AOP levels and SOD activity showed no statistically significant differences between the experimental groups and the control group.

**Figure 1:**
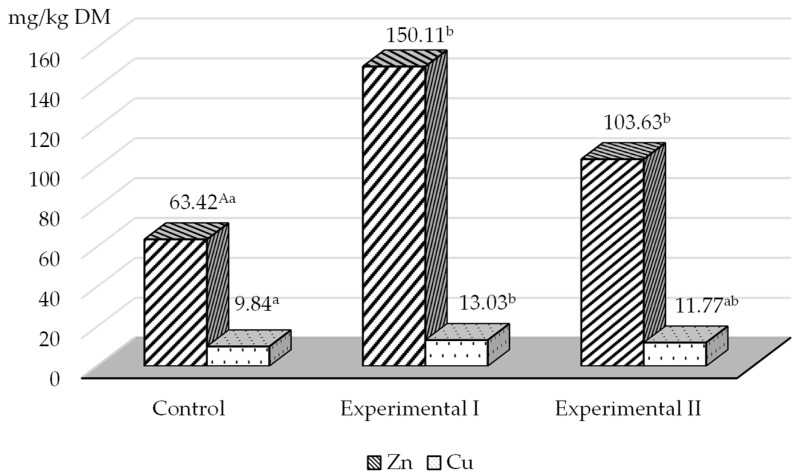
Mean values of zinc and copper content in the liver of 6-week-old chickens (mg/kg DM); ^a,b^—significant differences between groups at *p* ≤ 0.05; ^A^—significant differences between groups at *p* ≤ 0.01.

The highest content of Zn and Cu was found in the livers of chickens from experimental group I (150.11 mg/kg and 13.03 mg/kg, respectively), followed by the livers of chickens from experimental group II (103.63 mg/kg and 11.77 mg/kg, respectively), and the lowest in the livers of chickens from the control group (63.42 mg/kg for zinc and 9.84 mg/kg for copper). Regarding Zn content, the differences obtained were statistically confirmed at *p* ≤ 0.01 between experimental group I and the control group, and between experimental group II and the control group at *p* ≤ 0.05. Regarding Cu content in the liver, statistically confirmed differences (at *p* ≤ 0.05) were obtained between experimental group I and the control group. The results obtained indicate significantly better bioavailability of the tested elements when administered to birds in the form of hydroxychlorides (Figure 1).

**Figure 2:**
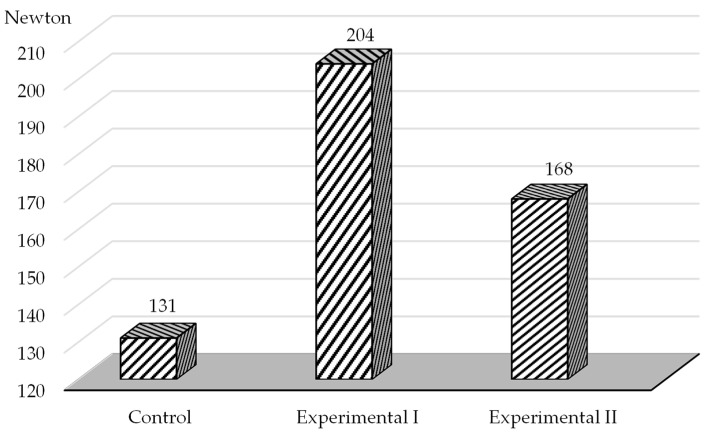
Average maximum load at tibial bone fracture (N).

Bone strength, expressed by the breaking force value, indicates a significant influence of the form of microelements used. The value of the examined trait was lowest in the case of birds fed with inorganic forms (control group) compared to the bone strength of chickens fed with Zn and Cu in the form of hydroxychlorides, which was 35% higher in experimental group I and 17% higher in experimental group II. The results obtained indicate the influence of the form of microelement administration on the degree of bone mineralization and, consequently, on their mechanical strength (Figure 2).

## 4. Discussion

Numerous studies on poultry nutrition show that the administration of so-called microelements during rearing has a significant impact on production indices [32]. Above all, the microelements Zn and Cu play an important role in the proper functioning of the organism [33,34,35,36,37]. The final BW obtained by Ross 308 broilers in our findings was lower than that obtained by Budnik [32], but higher than that obtained in studies by other authors [38,39]. Many studies point to the varied influence of the form and quantity of the discussed elements on the BW of chickens. Star et al. [40] and Gonzales-Esguerra et al. [41] did not find a positive effect of the form and level of Zn and Cu on increasing the final BW of chickens, while Budnik [32], Ethridge et al. [36] and Wijayanti et al. [42] obtained different results. Research by Zhao et al. [35] indicated the influence of the genetic line (Cobb 700 and Ross 708) on BW depending on the form (inorganic—sulfate and organic—chelate), which was partially confirmed in our findings.

In our findings, no significant influence of the Zn and Cu form administered to the birds on feed consumption was found. The influence on the FCR depending on the form and level of Zn and Cu in the dose was demonstrated by Ethridge et al. [36] and Gonzales-Esguerra et al. [41] when using the aforementioned elements in inorganic and organic forms in chicken nutrition. Wijayanti et al. [42] and Das et al. [43] found that replacing inorganic CuSO_4_ with Cu proteinate in the diet of broiler chickens positively influenced the feed utilization index.

Budnik [32] research indicates that the slaughter yield of broiler chickens of various lines ranges from 71.0 to 75.0%. Despite quite significant diversification in the examined slaughter traits of the chickens, a similar slaughter yield was found in Ross 308 chickens, ranging from 76.09 to 76.76%. Gheisari et al. [44] showed that broilers fed a mixture with the addition of Zn in the form of sulfate had a higher slaughter yield than those fed ZnO. The influence of the Zn and Cu forms administered to Cobb 700 and Ross 708 chickens in complete feed mixtures was demonstrated by Zhao et al. [35], stating a more favorable influence of organic forms of these elements on the increase in slaughter yield.

The most valuable part of the carcass is the muscle tissue. Depending on the applied form of Zn and Cu in the mixtures, carcasses differing in weight and the content of individual tissue components were obtained. Significantly lighter carcasses of chickens from the control group, fed with mixtures containing the inorganic form of Zn and Cu (zinc oxide and copper sulfate pentahydrate), were characterized by a better tissue composition than the carcasses of chickens from experimental groups I and II. This was expressed by clearly better muscularity, similar fatness, and a significantly lower share of the remainder of the carcass. On the other hand, the carcasses of chickens from the experimental groups were characterized by a similar share of tissue components; however, chickens from experimental group II, receiving Zn and Cu in mixtures in both inorganic and hydroxychloride form, compared to chickens from experimental group I, were characterized by slightly better muscularity and a lower share of the remainder of the carcass. Studies by other authors regarding Zn and Cu supplementation in doses for slaughter chickens suggest that the inclusion of organic Zn affects the increase in breast muscle weight and carcass muscularity [45]. In our findings, chickens from experimental group I, receiving microelements in the form of hydroxychlorides, were characterized by a highly significantly higher bone mass in the carcass compared to the control group and similar to experimental group II. The observed differences suggest a significant influence of the form of administered microelements on the skeletal development of broiler chickens.

The research results showed that regardless of the form of microelements used, the breast and leg muscles of Ross 308 chickens were characterized by a similar composition, showing similar values for dry matter, crude protein, and crude ash. However, a significant diversification in crude fat content in the breast muscles was found. Chickens from the control group contained significantly less of this component compared to the breast muscles of chickens from both experimental groups, which may suggest an influence of the nutrition used (Zn and Cu forms in mixtures) on the fat content in the examined muscles. Research by Salim et al. [46] conducted on broiler chickens shows that various sources and doses of Zn (zinc oxide, zinc amino acid chelate, and zinc sulfate) influence the muscle composition and sensory properties of broiler chicken meat, which is confirmed in our findings. Furthermore, Bao et al. [47] demonstrated that Zn oxide can improve the meat quality of broiler chickens by increasing protein content and reducing fat content in the muscles.

Numerous research results indicate that high Cu levels also cause high Cu accumulation in the liver [19,20]. The highest Zn and Cu content was found in the livers of Ross 308 chickens from experimental group I, slightly lower in chickens from experimental group II, and the lowest in the livers of chickens from the control group. The results obtained thus indicate significantly better bioavailability of the tested elements when administered to birds in the form of hydroxychlorides.

Zinc plays a special role in the development of the skeletal system in chickens [48,49]. Research on the strength of the tibial bones of broiler chickens showed that the highest average value of this trait was found in chickens from experimental group I, slightly lower in experimental group II, and the lowest in the control group. The results obtained indicate that the form of microelement administration to chickens influences the degree of bone mineralization and, consequently, their mechanical strength. Suttle [50] suggests that Zn has a stimulating effect on mineralization, bone formation, and the preservation of bone mass, while research by Kwiecień and Winiarska-Mieczan [5] indicates that the organic form of Cu positively influences the biomechanical properties of femoral bones. On the other hand, Ao et al. [51] conclude that the period of Zn administration to birds plays a greater role in the content of this element in the tibial bones than the form itself. In earlier studies by this author, the influence of the level and form of Zn on the content of this element in the tibial bones was found, with the organic form proving more favorable for the birds [52].

Adding Zn and Cu in the form of hydroxychlorides to the diet can increase the antioxidant capacity of chickens [53,54,55]. These microelements act as antioxidants, neutralizing free radicals and protecting cells from oxidative stress, preventing damage to cells and tissues, which positively influences meat quality and bird health. In our findings, a decrease in DPPH activity was observed in the groups fed a mixture of microelements in the form of hydroxychlorides compared to the control group. A lower DPPH percentage indicates a reduction in the muscle’s natural capacity to scavenge free radicals. The research result is statistically significant, and the free radical “scavenging” effect dropped from 11% to 8%.

Glutathione (GSH) is a peptide consisting of three amino acids: cysteine, glutamine, and glycine. It is characterized by the presence of thiol groups, which give it antioxidant properties. It directly participates in the neutralization of the hydroxyl radical and singlet oxygen. Furthermore, it serves as a cofactor for antioxidant enzymes such as GPx and GST [56]. In our findings, the level of GSH, responsible for neutralizing oxygen-free radicals and preventing oxidative stress, showed significant differences between groups. An increase in GSH levels was found in the groups fed with hydroxychlorides compared to the control group. Experimental group I had a significantly higher GSH level compared to the control group and experimental group II. An elevated level of GSH in the organism causes an increase in the amount of GPx, which is confirmed by our findings.

Superoxide dismutase (SOD) enzymatic activity depends on the presence of Cu and Zn. While Cu is needed for the catalytic activity of SOD, Zn participates in proper protein folding and stability [57,58]. SOD plays an important role in initial protection against reactive oxygen species (ROS) and can catalyze endogenous antioxidant enzymes. Reducing the amount of ROS favors the improvement of meat storage quality [59]. In our findings, the assessment of the level of AOP and SOD activity did not reveal statistically significant differences between the experimental and control groups in both experiments on different commercial sets.

The research objective of this work assumed that the addition of highly absorbable forms of microelements to feed mixtures would influence the increase in certain enzymes neutralizing free radicals, which could contribute to reducing immunosuppression in animals. However, the observed lack of differences in SOD activity levels distinguishes our findings from the results of other authors [60,61], who reported a significant influence of the examined feed additives on the level of SOD in serum and selected tissues.

Research by Ghasemi et al. [62] shows that supplementing the feed mixture for Ross 308 chickens with chelates containing Fe, Zn, Cu, Mg, Se, and Cr improved the antioxidant parameters of blood serum and meat, regardless of the amount of chelate administered to the birds. Yaqoob et al. [63] demonstrated that simultaneous supplementation with glycine chelates of Cu, Zn, Fe, and Mn improved the antioxidant status of the liver and blood serum of laying hens compared to supplementation with these minerals in inorganic form.

Malondialdehyde (MDA), formed as a result of lipid peroxidation, is a highly reactive compound associated with oxidative stress, causing an increase in free radicals [64]. Higher antioxidant enzyme activity is a response to oxidative stress; often, a chronic stress factor contributes to a decrease in SOD activity and an increase in MDA content in the organism [65,66]. In our findings, it was proven that the concentration of MDA was significantly higher in experimental group I compared to the control group. The observed significant increase in the MDA level in the breast muscles of chickens from experimental group I suggests an intensification of cellular oxidative stress compared to the control group, which showed the lowest MDA concentration. It can be assumed that the combined action of Zn and Cu in hydroxychloride form contributed to the increase in MDA content, indicating lipid oxidation in the muscles.

## 5. Conclusions

In summary, the application of zinc and copper hydroxychlorides in broiler diets significantly enhances growth parameters, mineral bioavailability, and bone strength, while simultaneously lowering breast muscle slaughter yield and intensifying oxidative stress within these tissues.

## Figures and Tables

**Table 1:** Experimental design.

Specification	Group–Group Size–Feeding Period		
	Control	Experimental I	Experimental II
	75	75	75
Starter mixture with premix	Days 1–11		
	1	1	1
Grower 1 mixture with premix	Days 12–21		
	1	2	3
Grower 2 mixture with premix	Days 22–35		
	1	2	3
Finisher mixture with premix	Days 36–42		
	1	2	3

1—Zn and Cu in inorganic forms contained in the mineral-vitamin supplement. 2—Zn and Cu exclusively in hydroxychloride form. 3—Zn and Cu in both inorganic and hydroxychloride form.

**Table 2:** Chemical composition of mixtures used in broiler chicken rearing.

Specification	Type of Complete Feed Mixture			
	Starter	Grower 1	Grower 2	Finisher
Metabolic energy (kcal)	3010	3091	3158	3210
Fat (%)	4.61	4.97	5.77	5.84
Crude protein (%)	22.00	21.00	20.35	18.65
Methionine (%)	0.54	0.53	0.52	0.50
Methionine + cystine (%)	1.05	0.89	0.87	0.84
Lysine (%)	1.23	1.20	1.15	1.04
Tryptophan (%)	0.22	0.24	0.24	0.21
Threonine (%)	0.95	0.76	0.73	0.66
Digestible methionine (%)	0.50	0.50	0.49	0.47
Digestible methionine + cystine (%)	0.93	0.81	0.80	0.76
Digestible lysine (%)	1.25	1.08	1.04	0.93
Digestible threonine (%)	0.84	0.66	0.64	0.57
Digestible tryptophan (%)	0.20	0.21	0.20	0.19
Ash (%)	5.35	5.21	4.95	4.63
Calcium (%)	0.96	0.86	0.79	0.76
Total phosphorus (%)	0.52	0.51	0.49	0.44
Available phosphorus (%)	0.48	0.39	0.38	0.33
Cu (mg/kg)	16.00	24.09	24.02	23.35
Zn (mg/kg)	90.00	93.94	93.43	91.46
Na (%)	0.16	0.16	0.16	0.16
Cl (%)	0.23	0.29	0.29	0.27

**Table 3:** Addition of Cu and Zn forms to the mineral-vitamin premix used throughout the entire rearing period.

Specification	Unit	Group		
		Control ^1^	Experimental I ^2^	Experimental II ^3^
Cu (dicopper chloride trihydroxide) (3b409)	mg/kg	0	15.00	7.50
Cu (copper (II) sulfate pentahydrate) (3b405)	mg/kg	15.00	0	7.50
Zn (zinc hydroxychloride monohydrate) (3b609)	mg/kg	0	60.00	30.00
Zn (zinc oxide) (3b603)	mg/kg	60.00	0	30.00

^1^—Zn and Cu in inorganic forms contained in the mineral-vitamin supplement. ^2^—Zn and Cu exclusively in hydroxychloride form. ^3^—Zn and Cu in both inorganic and hydroxychloride form.

**Table 4:** Mean values ($\bar{x}$ in g) and standard deviation (SD) of the BW of broiler chickens during the rearing period.

Days of Rearing	Group ($\bar{x}$ ± SD)		
	Control ^1^	Experimental I ^2^	Experimental II ^3^
1	46.7 ^a^ ± 1.3	47.4 ^a^ ± 1.1	47.1 ^a^ ± 1.1
7	180 ^a^ ± 12.5	190 ^a^ ± 23.2	183 ^a^ ± 11.9
14	450 ^a^ ± 21.9	430 ^a^ ± 28.4	440 ^a^ ± 23.1
21	880 ^a^ ± 45.8	908 ^a^ ± 56.3	900 ^a^ ± 45.2
28	1310 ^a^ ± 67.8	1400 ^b^ ± 78.9	1370 ^a,b^ ± 39.7
35	1896 ^a^ ± 136.1	1950 ^a^ ± 122.9	1910 ^a^ ± 110.3
42	2590 ^a^ ± 134.8	2710 ^b^ ± 188.6	2690 ^b^ ± 124.4

^a,b^—significant differences between groups at *p* ≤ 0.05. ^1^—Zn and Cu in inorganic forms contained in the mineral-vitamin supplement. ^2^—Zn and Cu exclusively in hydroxychloride form. ^3^—Zn and Cu in both inorganic and hydroxychloride form.

**Table 5:** Mean values of the European Production Efficiency Factor (EPEF) of 6-week-old chickens.

Specification		Group		
		Control ^1^	Experimental I ^2^	Experimental II ^3^
Average final BW (kg)		2.83 ^a^ ± 0.06	2.98 ^b^ ± 0.05	2.90 ^a,b^ ± 0.05
Feed conversion ratio per 1 kg of BW (kg) (rearing days)	1–7	0.88 ^a^ ± 0.01	0.86 ^a^ ± 0.00	0.87 ^a^ ± 0.01
	1–14	1.08 ^a^ ± 0.01	1.10 ^b^ ± 0.00	1.10 ^b^ ± 0.00
	1–21	1.18 ^a^ ± 0.01	1.15 ^a,b^ ± 0.01	1.13 ^b^ ± 0.01
	1–28	1.31 ^a^ ± 0.02	1.29 ^a^ ± 0.01	1.30 ^a^ ± 0.01
	1–35	1.44 ^a^ ± 0.02	1.41 ^a^ ± 0.01	1.44 ^a^ ± 0.02
	1–42	1.61 ^a^ ± 0.02	1.55 ^b^ ± 0.02	1.57 ^a,b^ ± 0.02
Mortality for the entire rearing period (%)		3.29 ^a,b^ ± 0.41	3.51 ^a^ ± 0.38	3.14 ^b^ ± 0.22
European Production Efficiency Factor (EPEF)		397 ^a^ ± 21.50	428 ^b^ ± 19.00	416 ^a,b^ ± 18.40

^a,b^—significant differences between groups at *p* ≤ 0.05. ^1^—Zn and Cu in inorganic forms contained in the mineral-vitamin supplement. ^2^—Zn and Cu exclusively in hydroxychloride form. ^3^—Zn and Cu in both inorganic and hydroxychloride form.

**Table 6:** Mean values ($\bar{x}$ in g) and standard deviation (SD) of the slaughter analysis results of 6-week-old chickens.

Specification	Group ($\bar{x}$ ± SD)		
	Control ^1^	Experimental I ^2^	Experimental II ^3^
BW before slaughter (g)	2556.00 ^a^ ± 54.2	2679.00 ^b^ ± 53.1	2634.00 ^a,b^ ± 45.1
Total offal (g)	83.88 ^a^ ± 10.3	100.24 ^b^ ± 11.1	96.88 ^a,b^ ± 5.3
Total inedible viscera and slaughter waste (g)	509.92 ^a^ ± 26.7	540.36 ^a^ ± 45.8	526.31 ^a^ ± 44.1
Eviscerated carcass weight (g)	1962.20 ^a^ ± 97.9	2038.40 ^b^ ± 87.7	2010.09 ^b^ ± 111.3
Slaughter yield (%)	76.76 ^a^ ± 3.2	76.09 ^a^ ± 4.1	76.31 ^a^ ± 5.0

^a,b^—significant differences between groups at *p* ≤ 0.05. ^1^—Zn and Cu in inorganic forms contained in the mineral-vitamin supplement. ^2^—Zn and Cu exclusively in hydroxychloride form. ^3^—Zn and Cu in both inorganic and hydroxychloride form.

**Table 7:** Tissue composition of the carcass of 6-week-old broiler chickens ($\bar{x}$ in g) and standard deviation (SD).

Specification	Group ($\bar{x}$ ± SD)		
	Control ^1^	Experimental I ^2^	Experimental II ^3^
Carcass weight	1962.20 ^a^ ± 32.1	2038.40 ^b^ ± 22.2	2010.80 ^a,b^ ± 32.8
Superficial breast muscles	536.01 ^a^ ± 11.0	489.36 ^a^ ± 12.1	506.21 ^a^ ± 21.0
Deep breast muscles	99.73 ^a^ ± 4.7	85.48 ^b^ ± 10.1	84.35 ^b^ ± 4.6
Breast muscles	635.74 ^a^ ± 32.1	574.84 ^b^ ± 10.4	590.56 ^b^ ± 12.6
Thigh muscles	213.10 ^a^ ± 7.7	235.87 ^a^ ± 12.1	225.81 ^a^ ± 13.0
Drumstick muscles	227.24 ^a^ ± 11.1	212.17 ^a^ ± 6.7	220.10 ^a^ ± 8.7
Leg muscles	440.34 ^a^ ± 12.9	448.04 ^a^ ± 10.3	445.91 ^a^ ± 21.0
Skin with subcutaneous fat	173.86 ^a^ ± 12.1	161.13 ^b^ ± 12.8	165.64 ^b^ ± 9.5
Abdominal fat	21.06 ^a^ ± 2.3	27.38 ^a^ ± 1.9	25.31 ^a^ ± 2.2
Skin with subcutaneous and abdominal fat	194.92 ^a^ ± 20.3	188.51 ^a^ ± 14.3	190.95 ^a^ ± 11.3
Carcass remainder (bones and other muscles)	691.20 ^A,a^ ± 23.1	827.01 ^B,b^ ± 22.6	783.40 ^A,B,c^ ± 21.1

^a,b,c^—significant differences between groups at *p* ≤ 0.05. ^A,B^—significant differences between groups at *p* ≤ 0.01. ^1^—Zn and Cu in inorganic forms contained in the mineral-vitamin supplement. ^2^—Zn and Cu exclusively in hydroxychloride form. ^3^—Zn and Cu in both inorganic and hydroxychloride form.

**Table 8:** Share of tissue components in the eviscerated carcass weight of chickens (%).

Specification	Group ($\bar{x}$ ± SD)		
	Control ^1^	Experimental I ^2^	Experimental II ^3^
Carcass weight	100.00	100.00	100.00
Breast muscles	32.39 ^a^ ± 5.32	28.20 ^b^ ± 3.11	29.37 ^b^ ± 3.76
Leg muscles	22.44 ^a^ ± 2.08	21.98 ^a^ ± 2.17	22.18 ^a^ ± 3.05
Breast and leg muscles	54.83 ^a^ ± 7.34	50.18 ^b^ ± 5.54	51.55 ^b^ ± 4.98
Skin with subcutaneous fat and abdominal fat	9.93 ^a^ ± 1.23	9.25 ^a^ ± 1.55	9.50 ^a^ ± 1.02
Carcass remainder	35.23 ^A,a^ ± 2.87	40.57 ^B^ ± 4.31	39.00 ^b^ ± 4.01

^a,b^—significant differences between groups at *p* ≤ 0.05. ^A,B^—significant differences between groups at *p* ≤ 0.01. ^1^—Zn and Cu in inorganic forms contained in the mineral-vitamin supplement. ^2^—Zn and Cu exclusively in hydroxychloride form. ^3^—Zn and Cu in both inorganic and hydroxychloride form.

**Table 9:** Chemical composition of breast muscles of 6-week-old chickens (%).

Specification	Group ($\bar{x}$ ± SD)		
	Control ^1^	Experimental I ^2^	Experimental II ^3^
**Chemical composition of breast muscles**			
Dry matter	26.54 ^a^ ± 2.11	25.84 ^a^ ± 4.59	25.75 ^a^ ± 5.46
Crude ash	1.52 ^a^ ± 0.12	1.42 ^a^ ± 0.12	1.47 ^a^ ± 0.12
Crude protein	21.97 ^a^ ± 1.19	21.12 ^a^ ± 2.82	21.58 ^a^ ± 2.01
Crude fat	2.55 ^a^ ± 1.01	3.87 ^b^ ± 0.88	3.15 ^a,b^ ± 0.34
**Chemical composition of thigh and drumstick muscles**			
Dry matter	25.33 ^a^ ± 3.21	25.41 ^a^ ± 2.22	25.11 ^a^ ± 4.01
Crude ash	1.02 ^a^ ± 0.11	1.23 ^b^ ±0.08	1.13 ^a,b^ ± 1.02
Crude protein	19.23 ^a^ ± 1.11	19.57 ^a^ ± 1.23	19.85 ^a^ ± 0.92
Crude fat	4.84 ^a^ ± 0.34	5.55 ^b^ ±0.81	5.34 ^a,b^ ± 0.12

^a,b^—significant differences between groups at *p* ≤ 0.05. ^1^—Zn and Cu in inorganic forms contained in the mineral-vitamin supplement. ^2^—Zn and Cu exclusively in hydroxychloride form. ^3^—Zn and Cu in both inorganic and hydroxychloride form.

**Table 10:** Results of antioxidant enzyme activity and DPPH determinations in breast muscles.

Specification	Group ($\bar{x}$ ± SD)		
	Control ^1^	Experimental I ^2^	Experimental II ^3^
AOP [mM]	0.404 ^a^ ± 0.057	0.408 ^a^ ± 0.072	0.405 ^a^ ± 0.064
GPx [nmol/min/mL]	128.773 ^a^ ± 16.59	152.873 ^b^ ± 17.38	136.387 ^a^ ± 16.99
Carbonyl groups [nmol/mL]	34.499 ^a^ ± 10.09	45.282 ^b^ ± 10.80	39.394 ^a,b^ ± 10.31
SOD [U/mL]	5.330 ^a^ ± 0.64	5.575 ^a^ ± 0.43	5.410 ^a^ ± 0.49
DPPH [%]	75.739 ^a^ ± 0.64	69.075 ^b^ ± 0.43	72.412 ^a,b^ ± 0.55
GSH µM -SH	19.365 ^A,a^ ± 3.13	30.482 ^B,b^ ± 11.36	24.188 ^a,b^ ± 5.87
MDA [µM]	0.346 ^a^ ± 0.58	0.450 ^b^ ± 0.72	0.420 ^b^ ± 0.63

^a,b^—significant differences between groups at *p* ≤ 0.05. ^A,B^—significant differences between groups at *p* ≤ 0.01. ^1^—Zn and Cu in inorganic forms contained in the mineral-vitamin supplement. ^2^—Zn and Cu exclusively in hydroxychloride form. ^3^—Zn and Cu in both inorganic and hydroxychloride form.

## Data Availability

The original contributions presented in this study are included in the article. Further inquiries can be directed to the corresponding author.
